# Ⅰ期非小细胞肺癌的治疗：立体定向放疗能替代外科手术？

**DOI:** 10.3779/j.issn.1009-3419.2015.06.13

**Published:** 2015-06-20

**Authors:** 思愚 王

**Affiliations:** 510060 广州，中山大学肿瘤防治中心，华南肿瘤国家重点实验室 Sun Yat-sen University Cancer Center, State Key Laboratory of South China, Guangzhou 510060, China

*The Lancet Oncology*杂志2015年5月发表了一项由美国德克萨斯大学MD安德森癌症中心学者发起的研究——《立体定向放疗对比肺叶切除治疗可手术的Ⅰ期非小细胞肺癌（non-small cell lung cancer, NSCLC）：两项随机试验的汇总分析》^[1]^。分析结果显示，对于可手术的Ⅰ期NSCLC患者，立体定向放疗（stereotactic ablative radiotherapy, SABR）较外科手术可进一步提高患者的总生存。

这两项研究的对象为临床T1-2a（< 4 cm）、N0M0、可手术的NSCLC。按1:1比例随机分组，总共有58例患者入组，分别接受SABR治疗（31例）或肺叶切除加纵隔淋巴结清扫或取样（27例）。SABR组中位随访时间为40.2个月，手术治疗组中位随访时间为35.4个月。随访期间SABR组有1例患者死亡，而手术治疗组有6例患者死亡。3年总生存率在SABR组为95%，手术治疗组为79%（HR=0.14, *P*=0.037）。3年无复发生存率在SABR组为86%，在手术治疗组为80%（HR=0.69, *P*=0.54）。局部控制方面SABR组有1例患者局部复发、4例区域淋巴结复发；手术治疗组有1例区域淋巴结复发。

在耐受性方面，SABR组有3例患者发生3级治疗相关的不良事件，如胸壁疼痛、呼吸困难或咳嗽，1例出现乏力和肋骨骨折。未见患者发生4级事件或治疗相关的死亡。手术治疗组有12例患者出现3级-4级治疗相关的不良事件，包括出血、支气管残端瘘、贫血、体重减少、乏力、恶心和心率失常等。

可手术的Ⅰ期NSCLC的标准治疗是肺叶切除加纵隔淋巴结清扫或取样。在过去的10年间，SABR只在一些不可手术的Ⅰ期NSCLC患者中获得较好局部控制率^[2-4]^，其5年局部无复发率为10%。STARS研究和ROSEL研究是两项独立、随机的Ⅲ期临床研究，评估SABR在这类患者中的应用，但由于病例入组数增长缓慢，两项研究均提前中止。进而，研究者们对两项试验的数据进行了汇总分析，从以上结果看，SABR的疗效似乎优于外科手术，但尚存较多疑惑：

STARS临床研究原计划入组例数为1, 030例，最终入组31例，其研究终点为总生存；而ROSEL研究原计划入组960例，最终为27例，其研究终点为局部和区域控制率。由于两项研究入组缓慢，病例数均较少，而且原设计研究终点不一，所以其汇总分析的结果可靠性不够。局部控制率方面SABR组有1例患者局部复发、4例区域淋巴结复发；手术治疗组有1例区域淋巴结复发，说明外科手术对局部病变控制具有优势。

治疗前诊断方面，SABR组26%患者没有病理诊断，只有正电子发射型计算机断层显像（positron emission computed tomography, PET）-计算机断层扫描（computed tomography, CT）结果。我们知道PET-CT的阳性预测值只有80%左右。手术治疗组术后1例为良性肿瘤，1例由于疾病进展而未能手术。在例数少的情况下，SABR组出现2例为良性结节，将更明显地影响结果。

总分析的研究终点为总生存，两组中有7例患者出现死亡，其中手术治疗组6例，SABR组1例。但是，手术治疗组中非肺癌相关死亡为5例（第二原发癌1例，手术相关死亡4例），两组中肺癌相关死亡各1例。总生存的差异并非对肺癌的治疗效果不同所致，而是手术治疗组非肺癌相关死亡增加的结果。令人困惑的是，为何手术治疗组术后死亡率高达15%（4/27），是否与入组患者年龄偏大有关？

外科手术并发症较高，44%患者术后出现3级-4级毒副作用，手术治疗组27例患者中5例出现术后呼吸困难，2例肺感染。正如研究者所说手术治疗总生存率较低要归咎于手术影响了肺脏功能，进而使合并疾病加重。术前评估肺功能是判断患者可否手术的一个重要指标，术后较多患者出现呼吸困难说明术前肺功能评估不准，也就是说这些患者属于不可手术，应该选择SABR。但在解析手术治疗组3年总生存率优于既往其他研究时，作者解释为入组患者的病变较小、体能状态较好、术前分期更加准确、而且手术患者的合并疾病较少。似乎颇有矛盾。

因此，对于可手术Ⅰ期肺癌给予SABR治疗目前证据不足，外科切除仍然是可手术Ⅰ期肺癌的唯一标准治疗。
